# Comparison of Growth Velocity Among School Age Children With Different Body Mass Index From Childhood Into Early Adolescence in Hualien County, Taiwan: A Retrospective Cohort Study

**DOI:** 10.3389/fped.2021.599730

**Published:** 2021-02-12

**Authors:** Yu-Chao Hsiao, Jen-Hung Wang, Chia-Hsiang Chu, Yu-Hsun Chang, Jui-Shia Chen, Rong-Hwa Jan, Shang-Hsien Yang, Ming-Chun Chen, Wei-Chih Chou, Shao-Yin Chu, Pei-Chun Lai, Ching-Feng Cheng, Pin-Yun Chiu, Yu-Hsuan Liu, Yung-Chieh Chang

**Affiliations:** ^1^Department of Pediatrics, Hualien Tzu Chi Hospital, Buddhist Tzu Chi Medical Foundation, Hualien, Taiwan; ^2^Department of Medical Research, Hualien Tzu Chi Hospital, Buddhist Tzu Chi Medical Foundation, Hualien, Taiwan; ^3^School of Medicine, Tzu Chi University, Hualien, Taiwan; ^4^Department of Pediatrics, National Taiwan University Hospital, Taipei, Taiwan; ^5^Department of Medical Education, Evidence-Based Medicine Center, Hualien Tzu Chi Hospital, Buddhist Tzu Chi Medical Foundation, Hualien, Taiwan; ^6^Department of Pediatrics, Taipei Tzu Chi Hospital, Buddhist Tzu Chi Medical Foundation, Taipei, Taiwan

**Keywords:** obesity, puberty, growth velocity, body mass index, anthropometry

## Abstract

**Objective:** This study aimed to investigate the contribution of high body mass index (BMI) to growth velocity among school-aged children who remained in the same BMI categories for a 6-year period.

**Methods:** This retrospective cohort study included children who enrolled in the school year 2009 and remained in the same BMI categories during their 1st, 4th, and 7th grades (6–7, 9–10, 12–13 years of age). Annual linear growth velocity and weight gain were calculated and compared between sexes, BMI groups, and different times. Risk analysis and repeated measures analysis of variance were performed to identify the impact of BMI on growth velocity.

**Results:** Of the 1,637 subjects, 53.0% were male, and 2.5% and 10.9% belonged to BMI groups of overweight and obese, respectively. In students between 6 and 13 years of age, obesity was associated with higher annual weight gain and height gain. Risk analysis showed that obese subjects had higher linear growth velocity than normal BMI groups of both sexes between 6 and 9 years of age. Unexpectedly, overweight and obese girls between 9 and 13 years of age had less linear growth velocity than underweight girls at the same interval. Repeated measures analysis of variance in both sexes showed a significant statistical association between BMI and different times of growth. However, the effect was less in girls between 9 and 13 years of age.

**Conclusion:** Puberty may dominate over BMI as the main contributor to high growth velocity in girls with underweight BMI emerging into pubertal age.

## Introduction

Childhood obesity continues to be a significant public health concern globally in recent decades. In the United States, a report warned that the levels of severe obesity in all children aged 2–19 years and populations have increased in the past 18 years (1). Many studies have reported that the prevalence of severe obesity has increased quickly (2–4). In Taiwan, an increasing trend in overweight and obesity was observed among Taiwanese children and adolescents over a 2-year period (5). The Nutrition and Health Survey in Taiwan 2013–2016 report showed that the prevalence of childhood overweight and obesity among children aged 7–12 years was 28.4%. In Hualien County, the prevalence of overweight or obese was 25% among children aged 7–12 years after analyzing the dataset of the school year 2010 (6). In Taiwan, routine health examinations for students are a suitable way to identify health problems, such as obesity, early and refer them for further evaluation (6). Childhood obesity has increased the risk of adulthood obesity and its related complications, including cardiovascular and metabolic diseases, which will reduce quality of life (2). Associations of growth velocity and pubertal timing are possible predictors of health problems, including a higher risk of early cardiovascular and metabolic morbidity and mortality (5–7).

The worldwide pandemic of childhood obesity has increased interest in the relationship between obesity and growth patterns during childhood and adolescence. Growth velocity and puberty are associated with complex mechanisms that involve the neuroendocrine system, and obesity has a significant impact on the neuroendocrine system (7). Rapid weight gain in infants has been associated with a higher rate of childhood obesity, which has been associated with a higher risk of obesity in adulthood (8, 9). The number of adipocytes present in the body in adulthood is related to obesity and correlates with the rate of increase in adipocyte counts in childhood and adolescence (10). Increased risk of obesity-related complications is significantly associated with childhood obesity (11, 12). Thus, early intervention to prevent childhood obesity is needed (13).

Children with early-onset obesity seem to have higher growth velocity before puberty and earlier onset of puberty than normal-weight or underweight children (14). In 2014, De Leonibus et al. (15) showed that pre-pubertal obese boys and girls were predisposed to an early-onset of puberty and achievement of pubertal maturation. In addition, obesity has been associated with taller pre-pubertal stature, which is subsequently lost, leading to similar adult heights between obese and normal-weight children (15). Recent longitudinal studies in the United States (16) and Sweden (17) have observed inverse associations between high BMI for age in childhood and linear growth during adolescence. Furthermore, recent evidence has suggested a relationship between childhood obesity and the timing of pubertal milestones in boys and girls (18, 19), which is known to influence adolescent linear growth. In one study conducted in both Belarus and the United States, the authors supported the role of higher body mass index (BMI) in accelerating linear growth in early childhood (i.e., taller stature and longer trunk length in those aged 3–6 years), but earlier pubertal development and slower linear growth during adolescence (12–17 years of age) (11).

Thus, this study aimed to investigate the contribution of high BMI on growth velocity by retrospectively tracking multiple anthropometric measurements among children 6–13 years old who remained in the same BMI categories for a 6-year period. We hypothesized that obesity would contribute primarily and equally to high growth velocity in both sexes from childhood into early adolescence.

## Materials and Methods

### Subjects

The dataset in this retrospective cohort study was collected from the annual health examination records of school-aged children between 2009 and 2015 in Hualien County. The Hualien County Government Education Bureau reviewed and approved this study. This study was approved by the Research Ethics Committee of Hualien Tzu Chi Hospital for data review (REC No. IRB105-52-B). The students' parents or guardians provided written informed consent for participating in the health examinations.

### Measurement and Procedures

**Figure 1:**
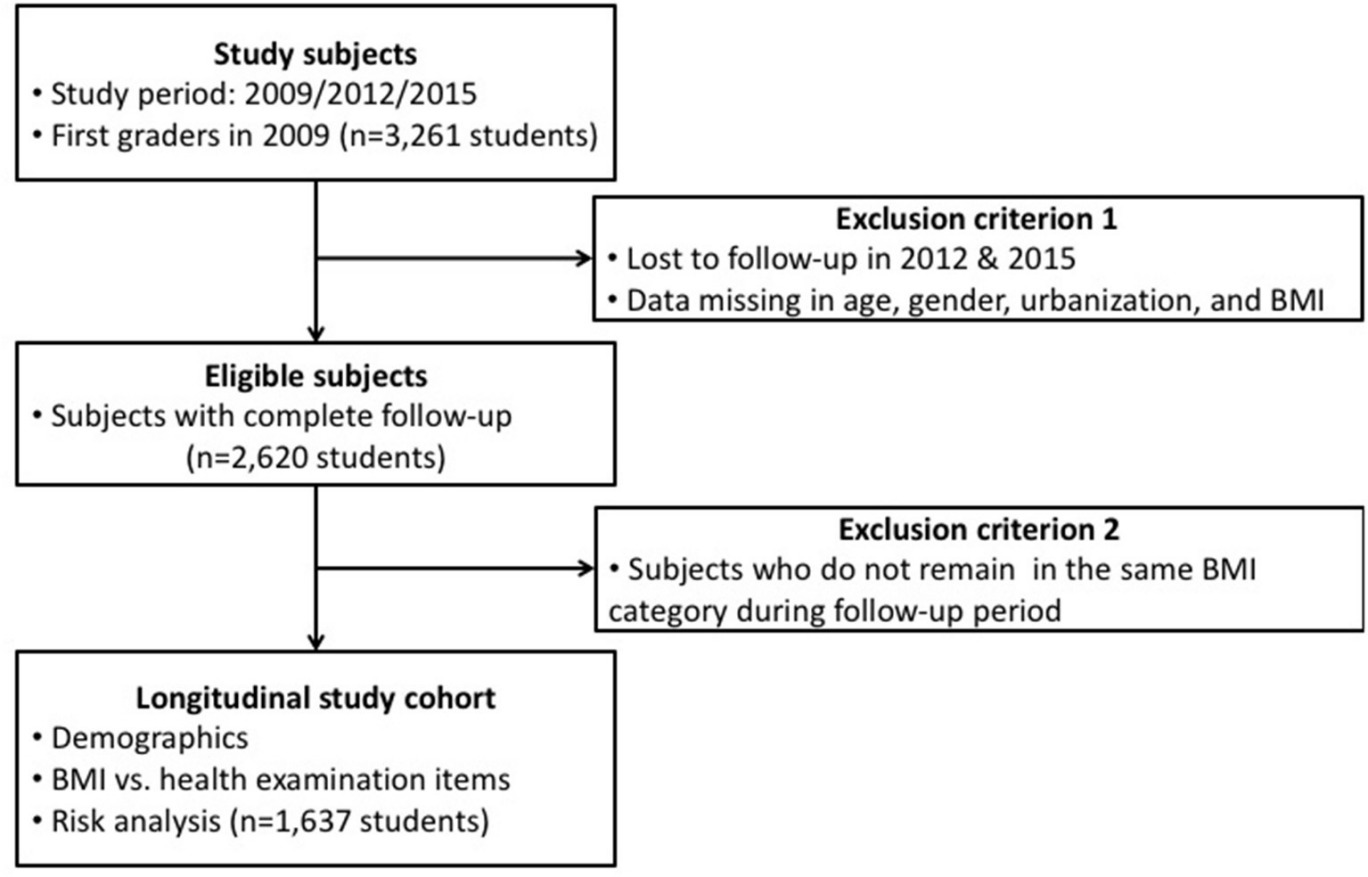
Flowchart of participant recruitment. BMI, body mass index.

Well-trained pediatricians, family physicians, and nurses from the medical center in Hualien County conducted health examinations of all students during their 1st, 4th, and 7th grade school years. Physical exam items recorded on student health examination records included eyes, teeth ears-nose-throat (ENT), head-neck, heart, chest-lung, abdomen, spine-limbs, male genitalia (hydrocele and varicocele), and skin systems according to the regulations set by the Taiwan Ministry of Education (20). Private parts such as female breasts and genitalia were not examined in school physical checkups. Tanner stages of both sexes were not evaluated and recorded. Anthropometrics including height, weight, and visual acuity were measured by school nurses prior to the physical examination dates. The portable stadiometer for height and weight measurement were mostly Seca digital column scale with integrated measuring rod (Seca, Germany) in school settings. Demographic data on health examination records included age, sex, and aboriginal and urban residential status, which was categorized into three groups: urban, suburban, and rural, according to the population densities of the residential areas (21). The flowchart of our study design is shown in Figure 1. Demographic data and anthropometric measurements of first graders enrolled in the 2009 school year, and their follow-up dataset as fourth (in 2012) and seventh (in 2015) graders were collected from the dataset of student health examination records. Students with missing data, such as age, sex, aboriginal and urban residential status, and BMI in 2012 and 2015 were excluded. Students with complete follow-up data were categorized into underweight, normal weight, overweight, and obese groups according to the age-sex-specific BMI cut-off values from the new growth charts for Taiwanese children and adolescents, based on the World Health Organization (WHO) standards, and health-related physical fitness, which was developed by the Department of Health in Taiwan in 2010 (6).

### Statistical Analysis

According to their BMIs, the students were categorized into four different groups: underweight, normal weight, overweight, and obesity groups (11). Annual linear growth velocity and weight gain were calculated and compared between sexes, BMI groups, and times. Multiple linear regression and repeated measures analysis of variance were performed to identify the impact of BMI on growth velocity since all subjects were examined three times during the follow-up period.

Descriptive statistics for sex, aboriginal and urban residential status are presented as frequencies or proportions. Annual height gain and annual weight gain are presented as mean ± standard deviation or median (lower quartile, upper quartile). The chi-square test was performed to evaluate the associations between categorical examination components and BMI groups. Analysis of variance was used to determine differences in means of continuous variables among different BMI groups. Multiple comparisons were performed using Bonferroni correction. Multiple linear regression models were used to simultaneously analyze the association between constant examination components and risk factors encountered.

Multiple logistic regression was performed to analyze categorical variables. Adjusted beta coefficients, odds ratios, and 95% confidence intervals were also calculated. Statistical significance was defined as *p* < 0.05. All statistical analyses were performed with Statistical Package for the Social Sciences version 17.0 (SPSS Inc., Chicago, IL, USA).

## Results

### Demographic Data

In total, 3,261 1st graders enrolled in the school year 2009 and their follow-up dataset as fourth graders (in 2012) and seventh graders (in 2015) were also collected. Two thousand six hundred twenty students who had complete follow-up data were categorized into underweight, normal weight, overweight, and obese groups according to the age-sex-specific BMI cut-off points from the new growth charts for Taiwanese children and adolescents as mentioned previously. Those 1,637 subjects who remained in the same BMI category over the 6-year period (2009–2015) were included for data analysis in the study (Figure 1). The demographic data including age, sex, urban residential status, aboriginal status, height, weight, and BMI are shown in Supplementary Tables 1, 2. The average height and weight among male and female groups of the same age did not show any significant difference (Supplementary Figure 1).

In Table 1, obesity was associated with higher annual weight gain (*p* < 0.001 in both sexes) and higher height gain in male students alone (*p* < 0.001 in male subjects and *p* = 0.09 in female subjects) between 6 and 13 years of age. There was no association between obesity and urban residential status.

**Table 1:** Demographics among BMI groups (2009–2015) (*N* = 1,637).

**Variable**	**Underweight**	**Normal**	**Overweight**	**Obese**	**Total**	***P*-value**	***Post-hoc* test**
**Male subjects (*****N*** **=** **867)**							
*N*	33	700	24	110	867		
Urbanization						0.084	
Urban, n(%)	29 (87.9)	511 (73.0)	17 (70.8)	82 (74.5)	639 (73.7)		
Suburban, n(%)	4 (12.1)	124 (17.7)	7 (29.2)	23 (20.9)	158 (18.2)		
Rural, n(%)	0 (0.0)	65 (9.3)	0 (0.0)	5 (4.5)	70 (8.1)		
Aborigine						0.025^*^	
No, n(%)	22 (91.7)	337 (68.9)	12 (80.0)	60 (78.9)	431 (71.4)		
Yes, n(%)	2 (8.3)	152 (31.1)	3 (20.0)	16 (21.1)	173 (28.6)		
**Female subjects (*****N*** **=** **770)**							
N	32	653	17	68	770		
Urbanization						0.712	
Urban, n(%)	24 (75.0)	467 (71.5)	14 (82.4)	48 (70.6)	553 (71.8)		
Suburban, n(%)	8 (25.0)	153 (23.4)	2 (11.8)	15 (22.1)	178 (23.1)		
Rural, n(%)	0 (0.0)	33 (5.1)	1 (5.9)	5 (7.4)	39 (5.1)		
Aborigine						0.262	
No, n(%)	22 (91.7)	333 (74.2)	10 (71.4)	38 (77.6)	403 (75.2)		
Yes, n(%)	2 (8.3)	116 (25.8)	4 (28.6)	11 (22.4)	133 (24.8)		
**Annual height gain (AHG) and Annual weight gain (AWG) of male subjects**							
AHG 1st−4th grade (mean ± SD, cm)	5.20 ± 0.63 5.13 (4.93, 5.50)	5.49 ± 0.76 5.50 (5.07, 5.93)	5.96 ± 0.64 5.93 (5.38, 6.49)	6.28 ± 0.73 6.25 (5.83, 6.78)	5.59 ± 0.80 5.60 (5.13, 6.03)	<0.001^*^	Obese, overweight > normal, underweight
AHG 4th−7th grade (mean ± SD, cm)	6.09 ± 1.45 5.93 (4.78, 7.20)	6.74 ± 1.47 6.60 (5.57, 7.90)	6.93 ± 1.25 7.12 (5.68, 8.16)	6.83 ± 1.03 6.80 (6.19, 7.51)	6.73 ± 1.42 6.67 (5.60, 7.87)	0.050	
AHG 1st−7th grade (mean ± SD, cm)	5.64 ± 0.84 5.53 (4.90, 6.10)	6.12 ± 0.87 6.08 (5.50, 6.80)	6.44 ± 0.80 6.66 (5.59, 6.98)	6.55 ± 0.69 6.53 (6.07, 7.08)	6.16 ± 0.87 6.17 (5.53, 6.83)	<0.001^*^	Obese, overweight > normal > underweight
AWG 1st−4th grade (mean ± SD, kg)	1.84 ± 0.37 1.87 (1.67, 2.05)	2.79 ± 0.82 2.63 (2.27, 3.23)	4.72 ± 0.78 4.45 (4.20, 5.38)	6.41 ± 1.34 6.32 (5.53, 7.38)	3.26 ± 1.54 2.77 (2.30, 3.77)	<0.001^*^	Obese > overweight > normal > underweight
AWG 4th−7th grade (mean ± SD, kg)	3.08 ± 1.01 2.87 (2.27, 3.77)	4.52 ± 1.30 4.43 (3.53, 5.43)	5.97 ± 1.08 5.82 (4.94, 6.87)	7.85 ± 2.21 7.65 (6.08, 9.31)	4.93 ± 1.85 4.70 (3.60, 5.83)	<0.001^*^	Obese > overweight > normal > underweight
AWG 1st-7th grade (mean ± SD, kg)	2.46 ± 0.58 2.38 (2.03, 2.78)	3.65 ± 0.86 3.61 (3.05, 4.25)	5.34 ± 0.69 5.33 (4.82, 5.86)	7.13 ± 1.47 6.84 (6.18, 8.06)	4.10 ± 1.54 3.78 (3.08, 4.70)	<0.001^*^	Obese > overweight > normal > underweight
**Annual height gain (AHG) and Annual weight gain (AWG) of female subjects**							
AHG 1st−4th grade (mean ± SD, cm)	5.48 ± 0.99 5.57 (5.26, 5.83)	5.95 ± 1.08 5.83 (5.33, 6.50)	6.87 ± 1.02 6.97 (6.05, 7.65)	6.87 ± 0.92 6.72 (6.33, 7.33)	6.03 ± 1.11 5.88 (5.37, 6.63)	<0.001^*^	Obese, overweight > normal, underweight
AHG 4th−7th grade (mean ± SD, cm)	6.54 ± 1.02 6.78 (5.53, 7.30)	6.33 ± 1.10 6.50 (5.73, 7.10)	5.47 ± 1.56 5.80 (4.43, 6.63)	5.07 ± 1.39 5.15 (4.21, 6.07)	6.21 ± 1.20 6.40 (5.57, 7.03)	<0.001^*^	Normal, underweight > obese, overweight
AHG 1st−7th grade (mean ± SD, cm)	6.01 ± 0.67 6.08 (5.76, 6.36)	6.14 ± 0.58 6.18 (5.83, 6.50)	6.17 ± 0.62 6.13 (5.83, 6.55)	5.97 ± 0.62 6.08 (5.53, 6.30)	6.12 ± 0.59 6.17 (5.82, 6.48)	0.090	
AWG 1st−4th grade (mean ± SD, kg)	1.96 ± 0.41 1.97 (1.65, 2.27)	2.95 ± 0.90 2.80 (2.30, 3.47)	5.12 ± 0.75 5.13 (4.52, 5.78)	6.55 ± 1.32 6.57 (5.54, 7.52)	3.28 ± 1.43 2.90 (2.33, 3.87)	<0.001^*^	Obese > overweight > normal > underweight
AWG 4th−7th grade (mean ± SD, kg)	3.52 ± 0.76 3.70 (2.88, 4.09)	4.58 ± 1.07 4.60 (3.90, 5.30)	5.18 ± 1.28 4.87 (4.00, 6.45)	7.19 ± 2.29 7.07 (5.45, 8.77)	4.78 ± 1.45 4.63 (3.90, 5.43)	<0.001^*^	Obese > overweight, normal > underweight
AWG 1st−7th grade (mean ± SD, kg)	2.74 ± 0.42 2.79 (2.53, 3.01)	3.77 ± 0.69 3.75 (3.30, 4.24)	5.15 ± 0.72 5.08 (4.56, 5.87)	6.87 ± 1.47 6.78 (5.68, 7.88)	4.03 ± 1.22 3.83 (3.30, 4.40)	<0.001^*^	Obese > overweight > normal > underweight

Values beneath mean ± SD are median (lower quartile, upper quartile).

* *p < 0.05 was considered statistically significant after test. AWG, annual weight gain; AHG, annual height gain; BMI, body mass index; SD, standard deviation*.

### Associated Factors With Physical Growth

Table 2 illustrates the factors associated with height gain and weight gain for male and female students, including BMI and different periods of growth (6–9 and 9–13 years of age). In female subjects aged 6–9 years, the growth showed higher linear growth velocity and weight gain in overweight and obese groups than in the normal BMI groups; at the same time, underweight girls had less linear growth velocity and weight gain than girls with normal BMI. Among female subjects between 9 and 13 years of age, overweight and obese girls had less linear growth than girls with normal BMI. Underweight boys between 6 and 13 years of age had less linear growth velocity and weight gain than boys with normal BMI. The obese boys between 6 and 9 years of age had higher linear growth velocity than boys with normal BMI. However, the overweight and obese boys aged 9–13 years did not show a significant difference in linear growth velocity compared to boys with normal BMI.

**Table 2:** Factors associated with height gain and weight gain.

	**AHG (6–9 years of age)**		**AHG (9–13 years of age)**		**AWG (6–9 years of age)**		**AWG (9–13 years of age)**	
**Variable**	**β (95% CI)**	***P*-value**	**β (95% CI)**	***P*-value**	**β (95% CI)**	***P*-value**	**β (95% CI)**	***P*-value**
**MALE STUDENTS (*****N*** **=** **867)**								
**BMI group^§^**								
Normal	Reference		Reference		Reference		Reference	
Underweight	−0.333 (−0.624, −0.043)	0.025^*^	−0.816 (−1.395, −0.237)	0.006^*^	−0.972 (−1.321, −0.622)	<0.001^*^	−1.365 (−1.953, −0.776)	<0.001^*^
Overweight	0.345 (−0.018, 0.708)	0.063	0.033 (−0.691, 0.757)	0.928	1.766 (1.328, 2.203)	<0.001^*^	1.405 (0.670, 2.140)	<0.001^*^
Obese	0.886 (0.715, 1.057)	<0.001^*^	0.196 (−0.144, 0.537)	0.258	3.805 (3.600, 4.011)	<0.001^*^	3.517 (3.171, 3.863)	<0.001^*^
**FEMALE STUDENTS (*****N*** **=** **770)**								
**BMI group^§^**								
Normal	Reference		Reference		Reference		Reference	
Underweight	−0.432 (−0.840, −0.024)	0.038^*^	0.170 (−0.289, 0.629)	0.468	−1.029 (−1.408, −0.650)	<0.001^*^	−1.152 (−1.658, −0.647)	<0.001^*^
Overweight	1.043 (0.516, 1.570)	<0.001^*^	−0.852 (−1.445, −0.259)	0.005^*^	2.222 (1.733, 2.710)	<0.001^*^	0.576 (−0.077, 1.228)	0.084
Obese	0.920 (0.628, 1.213)	<0.001^*^	−1.303 (−1.632, −0.974)	<0.001^*^	3.705 (3.434, 3.977)	<0.001^*^	2.828 (2.466, 3.190)	<0.001^*^

* P < 0.05 was considered statistically significant.

§ *Adjusted for aborigine status and urbanization. AWG, annual weight gain; AHG, annual height gain; CI, confidence interval; BMI, body mass index*.

**Figure 2:**
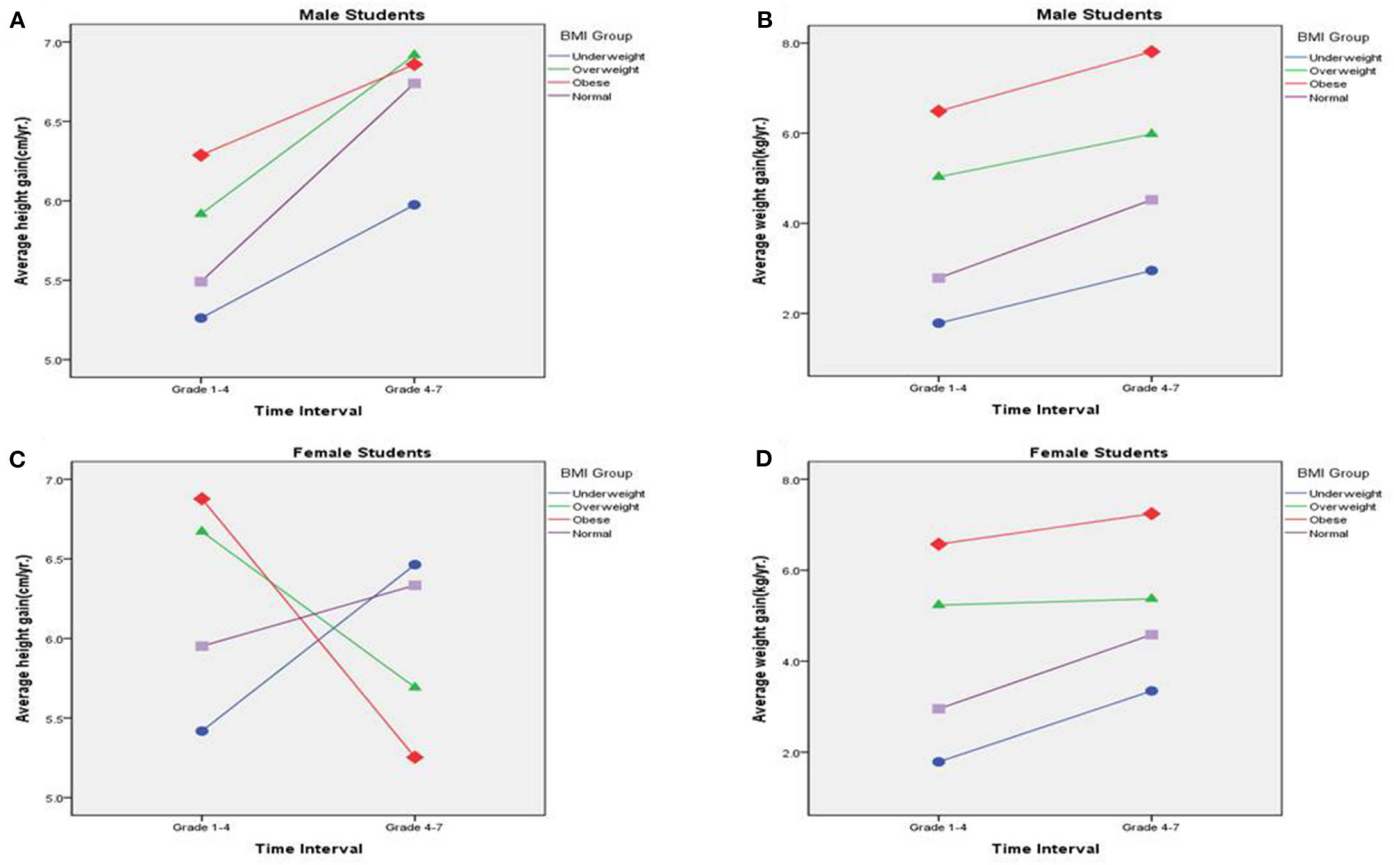
**(A–D)** Results of repeated measures analysis of variance for male and female subjects.

Supplementary Tables 3, 4 show the results of repeated measures analysis of variance for male and female subjects. In both sexes, a significant statistical association was observed between BMI and different times of growth (Figure 2). High BMI to the range of overweight and obese had strong effects on high linear growth in subjects between 6 and 13 years of age (1st to 7th grade); on the contrary, this effect was less in female subjects between 9 and 13 years of age (4th to 7th grade).

## Discussion

This study revealed that obese boys and girls had higher linear growth velocity between 6 and 9 years of age (1st to 4th grade) on risk analysis, but the finding was different in children aged 9–13 years (4th to 7th grade). The growth pattern among boys and girls was not the same—the obese and overweight girls had less linear growth velocity than underweight girls between 9 and 13 years old, which was not consistent with our hypothesis that obesity would contribute equally to high growth velocity in both sexes from childhood into early adolescence. It has been found that the average age of menarche in Taiwanese girls is about 12 years of age (general range, between 11 and 13 years of age) (22). Additionally, the age of female pubertal growth spurt was 2–3 years before menarche occurred. Obesity has also been related to the early onset of puberty including early menarche (19, 23, 24). Peripheral adipose tissue and interaction of leptin with the kisspeptin system may be associated with obesity and puberty (19). Many studies have found that the trend of obesity and early puberty onset is more prominent in girls than in boys (19, 23, 25, 26). Although we did not collect data on the onset of puberty or age of menarche, we speculated that overweight and obese girls in our study had an earlier onset of puberty and earlier age of menarche than normal and underweight BMI girls based on our clinical observation. Overweight and obese girls had earlier timing of growth spurt when compared with underweight BMI girls within the same age interval, between 9 and 13 years old, which may explain the low linear growth velocity of obese and overweight girls observed in the present study. However, the maximum growth velocity of boys was generally 2 years behind the growth spurt of girls, e.g., 10–12 years in girls vs. 12–14 years in boys.

Overweight and obese boys between 9 and 13 years of age did not show a significant difference in linear growth velocity when compared with boys with normal BMI. On the other hand, boys with underweight BMI between 6 and 13 years of age revealed less growth velocity than boys with normal BMI in the present study. Although overweight and obese boys between 6 and 9 years of age had significant high growth velocity compared to boys with normal BMI, high BMI did not have a strong impact on growth velocity in boys between 9 and 13 years of age when emerging into puberty. It is still controversial whether obesity has an impact on the early onset of puberty in boys (11). The present study illustrated that low BMI still reduced the growth velocity in underweight boys aged 9–13 years. However, longitudinal growth data from previous studies revealed that adult heights were not different among different BMI groups (17). In addition, taller standing heights during early adolescence (10–15 years of age) were observed among children with higher BMIs during mid-childhood (6–9 years of age). Furthermore, the greater heights in early adolescence were mainly attributable to longer trunks, rather than longer legs (11). De Leonibus et al. (15) reported that mean ages at the onset of pubertal boys were about 11.66 years in the obese group and 12.12 years in the normal-weight group. Similarly, late puberty was reached at a younger age in obese children. Mean ages at late puberty of boys were 13.33 years in obese children and 14.47 years in normal-weight children (15). Although the present study lacks data on the physical findings of Tanner stages in both sexes, we could still speculate that obese and overweight boys may have lower growth velocity than boys with normal or underweight BMI after 13 years of age because most of the obese and overweight boys would complete their growth spurt by then. More data on the growth velocity of male students after 13 years of age should be collected in the future to confirm this hypothesis.

Nutrition status and BMI have been associated with growth velocity, as well as weight and height in childhood, and obese subjects are taller than non-obese ones in childhood (11, 14, 27). Some possible mechanisms have been proposed as responsible for this association, and correlations of growth hormone-binding protein, fasting insulin level, and insulin-like growth factor (IGF)-1 level with BMI were mentioned in some studies (11, 14, 27–29). The regulation of the growth hormone (GH)–IGF-1 axis has also been associated with prepuberty BMI (29). GH secreted from the anterior pituitary gland not only promotes the growth of bone and muscle but also has an essential role in the regulation of lipids. GH stimulates the secretion of IGF-1, and both GH and IGF-1 have been proven to be related to linear growth (30). In obese children, decreased GH secretion has been noted (28). However, hyperinsulinemia due to obesity-related insulin resistance may also be related to elevated free IGF-1 levels in obese children. An increase in free IGF-1 level is thought to be associated with rapid linear growth and skeletal maturation (14, 31). Thus, the above mechanisms may explain our finding of higher linear growth velocity in pre-pubertal obese children aged 6–10 years of age.

In Taiwan, parents and grandparents are usually proud of the high linear growth velocity of their obese children, rather than of obesity itself. They have a strong belief in our traditional proverb “childhood obesity is not obese at all” and often seek medical assistance for their children late when obesity-related complications, such as hypertension and high blood sugar or cholesterol, occur; however, delayed intervention for obesity leads to more harm to health. To achieve good family health, physicians should engage the concerns of parents to empower their knowledge of healthy literacy in nutrition, exercise, and parenting skills. A family-based exercise plan, healthy diet plan, and structural daily schedule will provide a supportive environment for children after leaving the school campus. Reward options should not include snacks, sweetened beverages, or high calorie foods; instead, outdoor sports or tours are recommended to promote physical activity. Management of childhood obesity is progressive and age-dependent, and it should be initiated as early as possible (32, 33). Based on the report of the Commission on Ending Childhood Obesity by the WHO in 2016, comprehensive programs that promote healthy school environments, health and nutrition literacy, and physical activity among school-age children and adolescents should be established and assisted by family-based, multicomponent lifestyle weight management services (34).

## Limitations

This study has some limitations that need to be addressed. First, the retrospective data in our study were collected from the annual health examination dataset of elementary school students in Hualien County; we did not have information of the subjects outside the period evaluated. Thus, we did not know their physical measurements after birth, Tanner stage, and final height in adulthood. Second, many factors affect the linear growth velocity in addition to obesity, such as exercise, diet, underlying diseases, and parents' heights, which were not collected during the regular school health examinations in our dataset. Further studies should be conducted to evaluate the associations between these factors and growth velocity. Third, the timing of puberty onset is essential to evaluate the change of growth velocity, especially in subjects with different BMIs. They should have different times for the onset of puberty, but no reliable physical findings of puberty were recorded in the present study.

## Conclusions

We support current models that higher BMI accelerates linear growth in childhood, but slows linear growth during early adolescence. Our findings illustrated that obese subjects tended to have higher linear growth velocity than subjects with normal BMI. However, underweight girls had higher linear growth between 9 and 13 years of age, which coincided with the onset of puberty and a growth spurt. Thus, we propose that puberty dominates over BMI as the main contributor to high growth velocity in underwent early adolescent girls.

## Data Availability Statement

All data generated or analyzed during this study are included in this article.

## Ethics Statement

The studies involving human participants were reviewed and approved by Research Ethics Committee of Hualien Tzu Chi Hospital. Written informed consent to participate in this study was provided by the participants' legal guardian/next of kin.

## Author Contributions

C-HC, Y-HC, J-SC, R-HJ, S-HY, M-CC, S-YC, P-CL, C-FC, P-YC, and Y-HL contributed to data collection. J-HW and Y-CC analyzed and interpreted the subjects' data. Y-CH and Y-CC were major contributors to writing the manuscript. All authors read and approved the final manuscript.

## Conflict of Interest

The authors declare that the research was conducted in the absence of any commercial or financial relationships that could be construed as a potential conflict of interest.

## Abbreviations

BMI: body mass index
GH: growth hormone
IGF: insulin-like growth factor.
