# Supplementary material for: Comparison of Growth Velocity Among School Age Children With Different Body Mass Index From Childhood Into Early Adolescence in Hualien County, Taiwan: A Retrospective Cohort Study
Source: Front Pediatr. 2021 Feb 12;9:599730. doi: 10.3389/fped.2021.599730 (PMC7907168; doi:10.3389/fped.2021.599730)

Supplementary Table 1. Demographic characteristics of the subjects (N = 1,637)

Variable	2009 (6 to 7 years of age)
N	1,637
Age in 2009 (mean ± SD; median (lower quartile, upper quartile), y)	6.40 ± 0.50<br>6.00 (6.00, 7.00)
Sex	
Female, n (%)	770 (47.0)
Male, n (%)	867 (53.0)
Urbanization	
Urban, n (%)	1,192 (72.8)
Suburban, n (%)	336 (20.5)
Rural, n (%)	109 (6.7)
Aborigine	
No, n (%)	834 (73.2)
Yes, n (%)	306 (26.8)
BMI group	
Underweight, n (%)	65 (4.0)
Normal, n (%)	1,353 (82.7)
Overweight, n (%)	41 (2.5)
Obese, n (%)	178 (10.9)

SD = standard deviation

BMI= Body mass index

Supplementary Table 2. Demographic characteristics of the subjects (N=1,637)

Variable	2009 (6 to 7 years of age)	2012 (9 to 10 years of age)	2015 (12 to 13 years of age)
Overall height (cm)	117.59 ± 5.21<br>117.50 (114.00, 121.00)	134.99 ± 6.56<br>134.50 (130.35, 139.50)	154.45 ± 7.47<br>154.20 (149.50, 159.20)
Female height (cm)	117.28 ± 5.18<br>117.45 (114.00, 120.80)	135.38 ± 6.79<br>135.00 (130.70, 140.00)	154.02 ± 6.03<br>154.10 (150.10, 157.90)
Male height (cm)	117.87 ± 5.23<br>117.50 (114.10, 121.30)	134.65 ± 6.33<br>134.20 (130.00, 139.00)	154.84 ± 8.53<br>154.50 (148.50, 161.00)
Overall weight (kg)	22.23 ± 4.85<br>21.10 (19.30, 23.30)	32.04 ± 8.73<br>29.70 (26.70, 33.70)	46.62 ± 12.44<br>43.80 (39.15, 49.40)
Female weight (kg)	21.72 ± 4.56<br>20.85 (19.00, 22.92)	31.55 ± 8.24<br>29.55 (26.50, 33.40)	45.90 ± 11.07<br>43.50 (39.80, 48.40)
Male weight (kg)	22.68 ± 5.05<br>21.40 (19.70, 23.80)	32.47 ± 9.13<br>29.80 (27.00, 34.00)	47.26 ± 13.52<br>44.00 (38.80, 50.60)
BMI (kg/m^2)	15.97 ± 2.52<br>15.35 (14.61, 16.30)	17.39 ± 3.50<br>16.38 (15.36, 17.85)	19.37 ± 4.08<br>18.41 (17.02, 20.05)

Values are presented as mean ± standard deviation with median (lower quartile, upper quartile).

SD = standard deviation

BMI= Body mass index

Supplementary Table 3. Results of repeated measures analysis of variance in male subjects (N=867)

Variable	Source of Variation	df	SS	MS	F	P-value
Height gain	Between subjects	866	1298.471			
	Treatment	3	57.975	19.325	13.444	<0.001*
	Error subjects within treatment	863	1240.496	1.437		
	Within Subjects	866	1082.674			
	Time	1	80.988	80.988	71.520	<0.001*
	Treatment X Time	3	24.442	8.147	7.195	<0.001*
	Error	863	977.244	1.132		
	Total	866	2381.145			
Weight gain	Between subjects	866	4093.964			
	Treatment	3	2548.674	849.558	474.454	<0.001*
	Error subjects within treatment	863	1545.290	1.791		
	Within Subjects	866	1111.601			
	Time	1	194.680	194.680	185.147	<0.001*
	Treatment X Time	3	9.489	3.163	3.008	<0.001*
	Error	863	907.432	1.051		
	Total	866	5205.565			

*p-value <0.05

SS = sum of squares, MS = mean of squares, df = degree of freedom

Supplementary Table 4. Results of repeated measures analysis of variance in female subjects (N=770)

Variable	Source of Variation	df	SS	MS	F	P-value
Height gain	Between subjects	769	540.357			
	Treatment	3	4.557	1.519	2.172	0.090
	Error subjects within treatment	766	535.800	0.699		
	Within Subjects	769	1517.502			
	Time	1	14.560	14.560	8.434	0.004*
	Treatment X Time	3	180.511	60.170	34.853	<0.001*
	Error	766	1322.431	1.726		
	Total	769	2057.859			
Weight gain	Between subjects	769	2271.733			
	Treatment	3	1336.727	445.576	365.036	<0.001*
	Error subjects within treatment	766	935.006	1.221		
	Within Subjects	769	989.191			
	Time	1	71.002	71.002	62.557	<0.001*
	Treatment X Time	3	48.776	16.259	14.325	<0.001*
	Error	766	869.413	1.135		
	Total	769	3260.924			

*p-value <0.05

SS = sum of squares, MS = mean of squares, df = degree of freedom

Supplementary figure 1: Physical measurements and change between the male and female groups

(A) Average height in 6 to7-year-old, 9 to10-year-old, and 12 to13-year-old

(B) Annual height gain in 6 to 9-year-old, 9 to 13-year-old, and 6 to 13-year-old

(C) Average weight in 6 to7-year-old, 9 to10-year-old, and 12 to13-year-old

(D) Annual weight gain in 6 to 9-year-old, 9 to 13-year-old, and 6 to 13-year-old

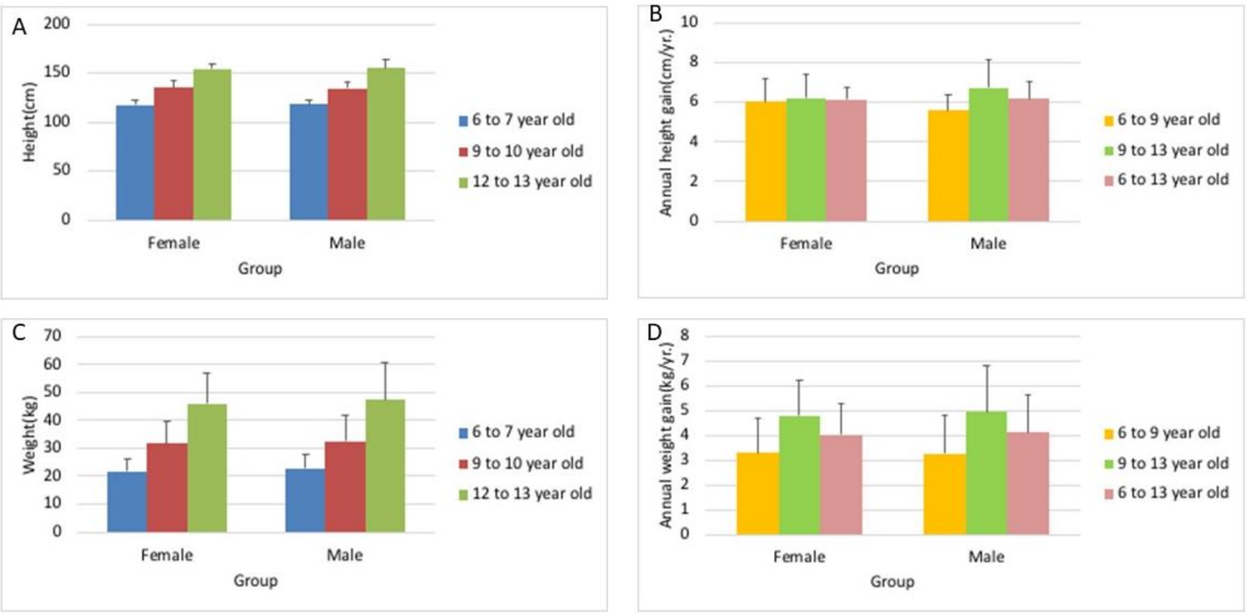

Supplement: Supplementary file 1 [file Data_Sheet_1.PDF]
